# Assessing Attitudes and Perceptions of High-Risk, Low-Resource Communities Towards Cardiopulmonary Resuscitation and Public-Access Defibrillation

**DOI:** 10.3390/jcm14020537

**Published:** 2025-01-16

**Authors:** Carolyn Hirsch, Bhanvi Sachdeva, Dilenny Roca-Dominguez, Jordan Foster, Kellie Bryant, Nancy Gautier-Matos, Mara Minguez, Olajide Williams, Mitchell S. V. Elkind, Shunichi Homma, Rafael Lantigua, Sachin Agarwal

**Affiliations:** 1 Office of Community Service Programs, Columbia University, New York, NY 10032, USA; 2 Mailman School of Public Health, Columbia University, New York, NY 10032, USA; bs3516@cumc.columbia.edu (B.S.);; 3 Department of Emergency Medicine, Columbia University Irving Medical Center, New York, NY 10032, USA; 4 National League for Nursing, Washington, DC 20037, USA; 5 School of Nursing, Columbia University, New York, NY 10032, USA; 6 NewYork-Presbyterian Hospital, New York, NY 10032, USA; 7 Department of Neurology, Columbia University Irving Medical Center, New York, NY 10032, USA; 8 The American Heart Association, Dallas, TX 75231, USA; 9 Department of Medicine, Columbia University Irving Medical Center, New York, NY 10032, USA

**Keywords:** cardiopulmonary resuscitation, health disparities, community development, education, community-based education

## Abstract

**Background:** Layperson cardiopulmonary resuscitation (CPR) and automated external defibrillator (AED) use are vital for improving survival rates after out-of-hospital cardiac arrest (OHCA), yet their application varies by community demographics. We evaluated the concerns and factors influencing willingness to perform CPR and use AEDs among laypersons in high-risk, low-resource communities. **Methods:** From April 2022 to March 2024, laypersons in Northern Manhattan’s Community District 12 completed surveys assessing their attitudes toward CPR and AED use before attending Hands-Only CPR training. Fisher’s Exact Test assessed differences in concerns and willingness to perform CPR and AED use across racial-ethnic groups and compared low-resource communities with high-resource groups consisting of non-clinical staff across eight ambulatory sites. **Results:** Among 669 participants from low-resource communities, 64% identified as Hispanic, 58% were under 40 years of age, and 67% were female. Significant knowledge gaps were identified: 62% had never learned CPR, and 77% were unfamiliar with AEDs. Top concerns about giving CPR included fear of incorrect performance (67%), causing harm (56%), and legal repercussions (53%). Willingness to perform CPR was most influenced by familiarity with the victim. The primary barrier to AED use was a lack of operational knowledge (66%). Non-Hispanic Black participants expressed significantly greater concerns than their Non-Hispanic White counterparts. Participants in high-resource settings (n = 309) showed higher training rates, albeit with similar apprehensions about CPR and AED use. **Conclusions:** Concerns regarding CPR and AED use stem from a lack of confidence and training, particularly predominant in certain racial-ethnic groups. Targeted, culturally sensitive community interventions could potentially address these barriers, enhance preparedness, and improve OHCA survival rates.

## 1. Introduction

Despite advancements in post-cardiac arrest care, survival rates following out-of-hospital cardiac arrest (OHCA) remain alarmingly low and vary significantly by neighborhood demographics [1,2]. For instance, New York City, with its diverse communities, reported survival rates ranging from 1.4% to 2.2% [3,4], starkly lower than the national average of 10–11% [5]. These differences are also reflected in their overall rates of sustained return of spontaneous circulation (ROSC) after OHCA (25% in New York City vs. 30% nationally) [6]. A critical determinant of survival and sustained ROSC is the prompt provision of cardiopulmonary resuscitation (CPR) and automated external defibrillator (AED) by laypersons, which can substantially improve survival chances [7,8]; however, only about 40% of OHCA victims receive immediate layperson CPR, and merely 4.6% receive defibrillation before emergency medical services arrive [9,10,11]. This phenomenon was exacerbated during the COVID-19 pandemic, where despite more OHCA occurring at home and being witnessed, the receipt of layperson CPR was significantly lower compared with pre-pandemic rates [12].

This low rate of OHCA lifesaving interventions is influenced by various contextual factors, including geographic, socioeconomic, and demographic characteristics of both the person experiencing OHCA and any layperson witness. Layperson CPR rates are notably lower in socially and economically disadvantaged areas, rural regions, and among certain ethnic populations, reflecting significant geographic and demographic disparities [13,14,15]. Individuals with lower socioeconomic status and Black and Hispanic individuals experience a higher incidence of OHCA [16,17] and reduced survival [18]. Black and Hispanic individuals are 37% less likely to receive lifesaving CPR in public spaces compared with their white counterparts [19].

These disparities further extend to the rates of CPR training across the United States [20]. Addressing the factors that influence a layperson’s willingness to perform CPR is essential for enhancing early resuscitation practices, improving survival rates, and reducing inequalities in high-risk, low-resource communities.

A layperson’s fear of causing harm and performing CPR incorrectly due to a lack of sufficient training has been identified as a major concern and significantly reduces confidence and the likelihood of providing CPR [15,21]. Notably, this lack of confidence has been closely linked to lower socioeconomic status and racial/ethnic composition of the community [9,13,14,22,23]. Additional fears of legal repercussions and personal safety, including the risk of contracting infections or diseases and being in potentially unsafe environments, have collectively contributed to the hesitancy and low rates of CPR initiation, particularly in vulnerable low-resource communities [13,14,15,24].

The willingness and likelihood of performing CPR during OHCA are significantly influenced by the relationship between the layperson and the individual experiencing it [25,26,27,28]. This reluctance to assist strangers is often attributed to concerns about the unknown disease status of strangers and the potential legal consequences of performing CPR [22]. In predominantly Hispanic/Latino neighborhoods, factors such as the mismatched age [26,29] and sex [30,31,32] of a victim compared with the layperson can decrease the likelihood of intervention. Cultural and social factors, including language barriers and a lack of community connectedness, could create reluctance to provide aid. These factors are particularly relevant in minority and economically disadvantaged neighborhoods, where such issues are more prevalent [23,33]. These challenges underscore the need for targeted community interventions that address both relational and demographic factors to increase the rates of laypersons’ willingness to perform CPR.

A recent systematic review and meta-analysis involving 1,081,040 OHCA patients across 11 countries has highlighted the significant benefits of community-based interventions across all populations [34]. These interventions have been associated with increased rates of layperson-initiated CPR, higher utilization of AEDs, improved survival rates, and better neurological outcomes among survivors [34]. Despite these promising findings, effectively addressing barriers, concerns, and misconceptions about these interventions requires a deep understanding of community attitudes and perceptions regarding CPR and AED use [35].

To address this knowledge gap, this study aimed to achieve three primary objectives: First, to evaluate the knowledge and training gaps, as well as the attitudes and perceptions that may influence the willingness of laypersons in high-risk, low-resource communities in New York City to perform CPR and use AEDs. Second, to identify specific racial and ethnic differences in attitudes and perceptions to inform the development of culturally appropriate community intervention programs. Finally, to compare findings from low-resource communities with those of non-clinical staff working in high-risk, high-resource outpatient medical facilities.

## 2. Methods

### 2.1. Study Design

We used a prospective anonymous survey design to determine layperson attitudes and perceptions contributing to willingness to perform Hands-Only CPR and AED use. This study protocol was initially approved by the Columbia University Institutional Review Board on 4 September 2018 (AAAR8497).

### 2.2. Study Population

#### 2.2.1. Low-Resource, High-Risk Community

Between 1 April 2022 and 31 March 2024, 669 laypersons across 20 groups voluntarily participated in in-person Hands-Only CPR and AED training sessions and filled out a survey as part of a community-based initiative, Community Development–Resuscitation Education, AED, and CPR Training (CD-REACT) [36]. The groups included community-based social service organizations (45%), youth extracurricular programs (26%), parent-teacher associations (19%), and local businesses (10%). Columbia University’s Office of community affairs representatives joined local organizations’ community board meetings to create awareness of the availability of CPR/AED training. In addition, several organizations volunteered to participate in the training after learning about the program via emails from the office of community affairs, in-person announcements, and flyer distribution in English and Spanish. Participants were also recruited from ongoing initiatives offered by the institutional programs for youth STEM, fitness and health education programs for older adults, community research forums, community health fairs, and longitudinal community-based participatory research studies.

The participants in this study were residents of Northern Manhattan’s Community District 12, which includes the neighborhoods of Washington Heights–Inwood, as well as parts of the Bronx and Harlem. With high area deprivation indices of these neighborhoods (7–10 deciles at the state level and 71–100 percentiles nationally) [37], they rank among the most socioeconomically disadvantaged in New York State and the country. Washington Heights–Inwood, the primary recruitment area, is characterized by a high-risk, low-resource population, with 68% identifying as Hispanic, 20% as Non-Hispanic White, and 33% with limited English proficiency [38]. Compared with other New York City neighborhoods, Northern Manhattan residents have lower high school graduation rates, higher poverty rates (20% below the federal poverty line), and greater feelings of unsafety in their neighborhoods [39]. Additionally, heart disease is the leading cause of death and hospitalization in this area, which ranks among the bottom 10 of 41 NYC neighborhoods for access to medical care [39].

#### 2.2.2. High-Resource, High-Risk Community as a Comparison Group

From October 2018 to May 2019, the Columbia University Patient Safety Department conducted in-person training sessions covering all major outpatient medical and surgical specialties (~1 million patient visits/year) throughout the New York Metro area. The outpatient medical sites included in this study rely on the emergency medical system for managing acute cardiac conditions, requiring clinical and non-clinical staff to assume the role of first responders in providing CPR and AED administration. Approximately 80% of the non-clinical staff at eight ambulatory sites receiving training constituted the high-risk, high-resource comparison group.

### 2.3. Training

The ≈1-h training session, in English or Spanish and identical for both low-and high-resource settings, was structured around three key components: Education, Demonstration, and Hands-on Skills Practice (see Appendix A for details on the training session).

Pre-Training Preparation:

Before the session began, participants completed a survey assessing their attitudes and perceptions of lifesaving interventions. Each participant received the American Heart Association (AHA; Dallas TX, USA) CPR Anytime Kit, which included a manikin for use during the training.

1. Education (15 min):

The session commenced with an educational segment. When audio-visual equipment was available, the AHA “CPR in Action” video was shown. This video was followed by an explanation of the differences between cardiac arrest and heart attack, the benefits of Hands-Only CPR, and the Chain of Survival’s role in keeping the brain and heart of the patient alive. Participants were encouraged to share any previous experiences with administering CPR.

2. Demonstration (10 min):

Next, a demonstration of Hands-Only CPR was conducted. This included a detailed overview of the technical aspects, such as the rate, depth, quality, and duration of effective CPR.

3. Hands-on Skills Practice (20 min):

Participants then engaged in Hands-on Skills Practice using their take-home CPR manikins. During this time, the training team circulated the room to assist with technique and answer any questions.

4. AED and Q&A (15 min):

The final segment covered the role of an AED in the Chain of Survival, common fears associated with AED use, and typical locations where AEDs are found. The use of the AED was demonstrated through active participant engagement. The session concluded with five minutes dedicated to addressing participant questions and concerns, such as what action to take if ribs are broken during CPR or the legal protections for performing CPR on a stranger.

### 2.4. Survey Instrument

We utilized a 16-item closed-question survey adapted from previous studies assessing attitudes and perceptions toward CPR/AED use in other populations [40]. Once developed, we ensured internal validity through testing within the five research team members and external validity by conducting a pilot test with 309 non-clinical staff members from outpatient sites. The survey, available in both English and Spanish, included demographic questions (such as age, sex, ethnicity, race, years of education, and current employment status) and inquiries about prior training in CPR and AED use. Participants were asked whether they believe cardiac arrest is the same as a heart attack, with response options of yes, no, or unsure. We also inquired about their awareness of the presence or location of an AED at their workplace or school. To gauge motivations for training, we provided several options categorized broadly into a general interest in learning, work requirements, or helping/saving a life in an emergency. Participants answered two primary questions with multiple choices regarding their general concerns about performing CPR and factors that might influence their willingness to perform CPR. They could select multiple options and add responses in an ‘Other’ category if desired. The final multiple-choice question asked for reasons they might not use an AED on a stranger who collapses in public (see Appendix B for the survey).

### 2.5. Data Analysis

Descriptive statistics (percentages and proportions) were first used to report the motivations, attitudes, and perceptions associated with general willingness to perform CPR and AED use. The ‘Other’ response, along with explanations, was analyzed by two independent research members to identify additional concerns or willingness factors; however, none of them achieved greater than 5% of the total responses to be included as a separate category. We then used Fisher-Exact and Chi-square tests to compare the differences between community members from high-resource and low-resource settings and the three racial-ethnic groups (Non-Hispanic White, Non-Hispanic Black, and Hispanic) for general concerns and factors associated with willingness to perform CPR.

Following this, we conducted both univariate and multivariate regression models to assess the associations between each concern and willingness factor (as outcomes) and the three racial-ethnic groups described above, with Non-Hispanic White participants serving as the reference group. These models were adjusted for key confounders, including participant age, sex, education, and prior CPR training status. *p*-value < 0.05 was considered statistically significant. Data were compiled and analyzed using STATA version 18.0.

## 3. Results

### 3.1. Low-Resource, High-Risk Community

#### 3.1.1. Demographics

Of the 669 laypersons who participated, 58% were under 40 years old. The majority identified as female (67%, n = 445) and Hispanic (64%, n = 426). Additionally, 24% (n = 163) identified as Black and 56% (n = 373) as White. Of the 163 Black participants, 46% (n = 75) were Non-Hispanic, while 23% (n = 84) of the 373 White participants identified as Non-Hispanic. While most participants (66%) had received high school or greater education, only 41% were employed at the time of the survey.

#### 3.1.2. Knowledge Gaps Regarding CPR

Most participants (65%) had either never learned CPR or were unsure about it, and over half (55%) responded either incorrectly or were unsure about cardiac arrest being the same as a heart attack (Table 1).

#### 3.1.3. Motivation for Training

**Figure 1:**
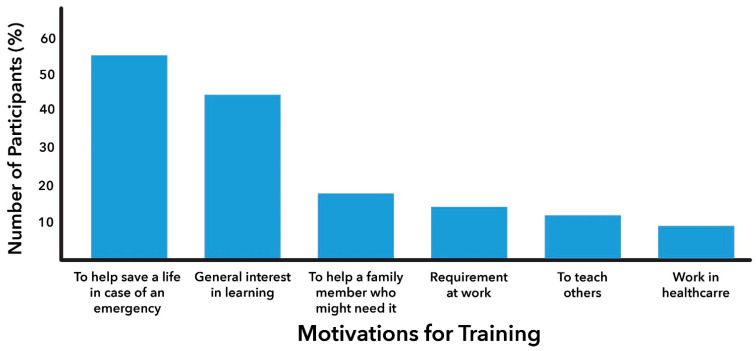
Motivations for CPR/AED Training Participation Among Laypersons in Low-Resource Communities.

When asked about their primary motivation for taking the course, the most frequently reported response was the desire to help save a life in an emergency, with 55% of participants (n = 361) citing this reason. This motivation was followed by a general interest in learning, reported by 43% of participants (n = 287). Other notable motivations included the ability to assist a child or an ill family member (18%; n = 122) and a work requirement (13%; n = 86) (Figure 1).

#### 3.1.4. General Concerns with Performing CPR

The primary concern among participants was the fear of performing CPR incorrectly due to insufficient training (67%, n = 423). This was followed by concerns about potentially harming the person experiencing OHCA (56%, n = 353). A close third concern was the fear of legal repercussions or lawsuits, particularly if the person were to die, reported in 53% of responses (n = 336) (Table 2).

#### 3.1.5. Factors Influencing Willingness to Perform CPR

The top two factors influencing the willingness to perform CPR were in relation to the familiarity with the person experiencing OHCA. Participants expressed more willingness to perform CPR on family members (55%, n = 325) or on someone they knew (67%, n = 398). The third most important factor was whether the layperson was a medical professional or not, reported in 34% of responses (n = 198). Among demographic characteristics, age was considered more frequently (34%, n = 203) compared with sex (20%, n = 120) or race/ethnicity (14%, n = 81) of the person experiencing OHCA (Table 3).

#### 3.1.6. Knowledge Gaps and Barriers Associated with the Use of Public Access Defibrillators

Significant learning gaps were identified, with 77% (n = 504) of participants reporting that they had never learned how to use an AED and an additional 6% (n = 37) being unsure. A majority (62%, n = 386) were unaware of whether an AED was available at their workplace or school, while 16% (n = 102) were certain that no AED was present (Table 1).

The primary barrier to public access to defibrillation was a lack of knowledge on how to use AEDs (66%, n = 417). Additionally, concerns about causing harm to the individual experiencing OHCA (25%, n = 158) and perceptions of difficulty in using an AED (17%, n = 108) were also commonly cited factors (Table 4).

#### 3.1.7. Stratified Analysis by Race-Ethnicities

##### Knowledge Gaps

Hispanic participants reported the lowest rate of previous CPR training (29%, n = 121), followed by Non-Hispanic Blacks (50%, n = 38) and Non-Hispanic Whites (63%, n = 53; *p* < 0.01). Similar disparities were seen in AED training, with the lowest rates among Hispanic (13%, n = 55) and Non-Hispanic Black participants (24%, n = 18), compared with Non-Hispanic Whites (39%, n = 33; *p* < 0.01).

Regarding whether cardiac arrest is the same as a heart attack, the majority of Hispanic (52%, n = 215) and Non-Hispanic Black (63%, n = 46) participants were either incorrect or unsure, compared with 53% (n = 42; *p* = 0.09) of Non-Hispanic Whites.

The ranking of the top three concerns, factors affecting the willingness to provide CPR, and barriers related to AED use were consistent across all racial-ethnic groups; however, Non-Hispanic Black participants expressed significantly higher concerns than Non-Hispanic White and Hispanic participants. Specifically, 79% of Non-Hispanic Black participants worried about performing CPR incorrectly (64% for both other groups, *p* = 0.03). Additionally, 75% feared harming the person in cardiac arrest (61% and 49%, *p* < 0.01), and 49% were concerned about contracting a disease (44% and 30%, *p* = 0.01). Last, 51% believed someone else would perform CPR better compared with 28% and 23% in the other groups (*p* < 0.01) (Table 2).

Compared with Non-Hispanic White, Non-Hispanic Black participants were significantly more likely to have confidence deficits evidenced by concern regarding performing CPR incorrectly (OR 2.5 (CI 1.2–5.2), *p* = 0.02), and that someone else will perform better (OR 3.2 (CI 1.6–6.5), *p* < 0.01), after adjustment for participant’s age, sex, education and prior CPR training status (Table 5).

Non-Hispanic Black participants were significantly more likely than Non-Hispanic White and Hispanic participants to indicate that certain factors influence the willingness to perform CPR. Notably, 82% of Non-Hispanic Black participants reported that knowing a person in some capacity (not including family members) affects their desire to act, compared with 70% of Non-Hispanic White and 63% of Hispanic participants (*p* < 0.01). There were no differences found across the three groups in willingness to perform CPR on a family member (57% vs. 54% vs. 56%, *p* = 0.9). Additionally, 22% of Non-Hispanic Black participants felt that the race-ethnicity of the person experiencing cardiac arrest would impact someone’s willingness to act, compared with 7% and 12% in the other groups (*p* = 0.03) (Table 3).

Compared with Non-Hispanic White, Non-Hispanic Black participants were significantly more likely to report that knowing a person in some capacity (OR 2.3 (1.0–5.3), *p* = 0.04), and the race-ethnicity of the person experiencing cardiac arrest would impact someone’s willingness to act (OR 3.7 (1.2–10.8), *p* = 0.02), after adjustment for participant’s age, sex, education and prior CPR training status (Table 6).

A significantly higher percentage of Non-Hispanic Black participants expressed barriers to using an AED compared with Non-Hispanic White and Hispanic participants. Most significant barriers included a belief that using an AED could harm the person in cardiac arrest (41% vs. 25% vs. 22%, *p* < 0.01), difficulty using an AED (29% vs. 20% vs. 13%, *p* < 0.01), or a feeling they were not allowed to use one (28% vs. 8% vs. 14%, *p* < 0.01) (Table 4).

### 3.2. Characterizing the High-Resource Community and Comparisons with the Low-Resource Community

#### 3.2.1. Demographics and Knowledge Gaps

Compared with participants from low-resource communities, non-clinical staff members from outpatient sites constituting the high-resource comparison group (n = 309) had significantly higher percentages of females (79% vs. 67%, *p* < 0.01) and a lower percentage of individuals of Hispanic ethnicity (32% vs. 64%, *p* < 0.01). Additionally, a significantly greater percentage of the high-resource group had previously learned CPR (60% vs. 38%, *p* < 0.01) and the use of an AED (32% vs. 17%, *p* < 0.01).

#### 3.2.2. Motivation for Training

Similar to participants from low-resource communities, the primary motivation for training among the participants in the high-resource group was a desire to help save a life in an emergency (43%, n = 114), followed by a general interest in learning (37%, n = 98).

#### 3.2.3. General Concerns with Performing CPR

The high-resource group, similar to their low-resource community members, cited performing CPR incorrectly (63%, n = 184) and the fear of legal repercussions (47%, n = 137) as their top concerns. There were no statistically significant differences between the two groups regarding any of their concerns (Table 2).

#### 3.2.4. General Willingness to Perform CPR

Although significantly lower than among participants from low-resource communities, knowing the patient in any capacity other than family (48% vs. 67%, *p* < 0.01) and whether the person was a family member (39% vs. 55%, *p* < 0.01) were still the top two factors identified among the high-resource group (Table 3).

## 4. Discussion

This study identified significant gaps in CPR-related knowledge and training, as well as highlighted critical concerns and factors influencing laypersons’ willingness to perform CPR and use AEDs, particularly in high-risk, low-resource, racially/ethnically diverse communities.

The majority of laypersons, who were predominantly young, female, and Hispanic, demonstrated significant gaps in CPR and AED knowledge. Specifically, 62% had never learned CPR, and 77% had not received AED training. These findings highlight a pressing need for increased educational efforts and resources. Despite more than half expressing helping save a life as their primary motivation, concerns, including fear of performing CPR incorrectly, causing harm, and potential legal repercussions, were highly prevalent. This finding is consistent with previous research [1,15,21,41,42] and is irrespective of CPR training status. Addressing these specific fears through improved educational materials as part of CPR training and including them in public awareness campaigns, such as those related to New York State’s Good Samaritan Law [43] and the federal Cardiac Arrest Survival Act [44], will help these communities cultivate a culture of empowerment.

This study also found that familiarity with the patient—whether familial or otherwise—significantly influenced the willingness to perform CPR. This aligns with other studies [1,35] reporting a higher willingness to assist family members compared with strangers. Acknowledging this aspect of ‘human nature’ is essential for informing strategies such as engagement activities (e.g., workshops, social gatherings, volunteer days) that foster interaction among community members. Creating platforms for sharing experiences and partnering with local organizations—such as schools, nonprofits, and businesses—can broaden outreach and resources, reinforcing a sense of community. Although not as commonly reported by the participants, age- [14] and sex-based [32] disparities in willingness to perform CPR have also been identified in other studies.

### 4.1. Disparities and Cultural Considerations

There are known geographic and ethnic disparities in CPR use. Hispanic and Black neighborhoods receive significantly lower rates of bystander CPR compared with predominantly White neighborhoods [1,45]. Qualitative studies have indicated that economically disadvantaged Black and Hispanic communities often experience reduced community connectedness, which can make individuals less likely to assist strangers. In this study, Non-Hispanic Black participants were significantly more likely to report responses that express deficits in their overall confidence in performing CPR and hold beliefs that the race/ethnicity of the person and the familiarity with the person experiencing CPR impact a layperson’s willingness to engage, compared with their Non-Hispanic White counterparts. Non-Hispanic Black participants also found AED use to be more challenging.

Of note, despite having the lowest rates of CPR and AED training and the highest knowledge deficits in this cohort, Hispanic participants appeared to be the most willing to perform CPR. This may be attributed to the unique characteristics of Northern Manhattan, a densely populated and closely-knit community with a high proportion of foreign-born residents (48%). In this area, Dominicans represent the largest group (62%), followed by Mexicans (10.5%) and Puerto Ricans (approximately 7%) [38]. Whether the strong community ties and cultural cohesion within these groups, combined with enhanced training opportunities, could empower individuals and further increase their willingness to perform CPR and use AEDs remains to be tested. Tailoring interventions to address specific concerns and leveraging cultural strengths has been shown to improve the effectiveness of community-based programs [34]. This is particularly important for high-risk neighborhoods, where they are also less likely to be trained in CPR [46,47].

### 4.2. Comparison with High-Resource Group

Unsurprisingly, non-clinical staff members from high-resource outpatient sites had higher rates of prior CPR and AED training. Despite this, the concerns about performing CPR incorrectly and legal consequences mirrored those of the low-resource lay participants, suggesting that such concerns are pervasive across different community settings and that CPR training must be strengthened and/or reinforced through refresher courses and hands-on practice to enhance both comfort and competency. Previous research shows that outpatient professionals, who are often in situations requiring CPR, feel underqualified or undertrained [48]. Studies comparing outcomes of OHCA in clinics versus other public locations have found no significant differences in bystander CPR rates, ROSC, hospital admissions, or survival to discharge [49].

### 4.3. Implications for Community Interventions and Academic–Community Partnerships

These data underscore the importance of designing culturally sensitive and contextually relevant interventions to improve CPR and AED training. In low-resource communities, where training gaps and high levels of concern are prevalent, efforts should focus on reducing barriers, building confidence, and enhancing access to training resources according to local community needs. The CD REACT program was part of a robust effort by Columbia University and the affiliated hospital system to build community health initiatives centered on community partnership with stakeholder needs at the forefront. Clinicians, public health researchers, and trainees served as health educators, extending their roles beyond usual clinical and research settings. Training sessions were held at community partner sites, such as schools and organizational headquarters, in the language requested (English, Spanish, or bilingual) and at the times they requested to meet communities where they are. These community collaborations may begin with academic medical center and hospital representatives joining local community meetings as was performed in a study with Manhattan Community Boards 9, 10, and 12 (Morningside Heights, Central Harlem, and Washington Heights–Inwood).

Additionally, combining community-based and health system interventions, including CPR and AED training, as well as dispatcher-assisted CPR, could have a synergistic effect [34]. Mass media campaigns and awareness initiatives may further contribute to a positive shift in attitudes towards resuscitation and increase bystander willingness to perform CPR [50].

### 4.4. Strengths

Several key strengths underscore this study’s unique contributions to the existing literature. The novelty of this study and its findings are manifold:

1. Collaboration Between Healthcare Institutions and Community Stakeholders: This study highlights the critical importance of building effective partnerships between healthcare institutions and community stakeholders. Central to the project were the partnerships among internal and external partners including various departments and schools within the academic medical center, including the schools of medicine, nursing, and public health, hospital, and community partners, including local middle and high schools. Such collaboration is a prerequisite for designing impactful community-based interventions. Researchers and community-based partners rely on population-based survey data to address health needs and to unravel the “backstory” behind racial/ethnic inequities in health access and outcomes [51]. Population health surveys are uniquely positioned to uplift marginalized populations and serve as tools for health system accountability, especially for those whose risks might otherwise remain obscured in aggregate data [51].
2. Multicultural and Multilingual Cohort: The cohort is predominantly Hispanic (64%), and 24% identify as Black, providing a representative multicultural and multilingual sample that mitigates coverage bias. While earlier studies [14,15] employed qualitative interviews to identify barriers and facilitators to CPR and AED use, this study represents the first and largest survey-based investigation assessing the prevalence of these themes in a broad, diverse population.
3. Comparison Between Low-and High-Resource Communities: To our knowledge, this is the first study to compare the attitudes and perceptions of lay community members in low-resource neighborhoods with those from high-resource settings. The findings indicate that confidence deficits and fears of legal repercussions are prevalent in both groups, suggesting that certain components of future training and intervention programs could potentially apply across all high-risk environments.
4. Post-Pandemic Perspective: This study is also unique in its evaluation of lay communities’ attitudes and perceptions in the post-COVID-19 era. The pandemic brought about significant logistical and behavioral changes, making it essential to reassess community barriers before designing and testing community-based interventions. Understanding the current climate of laypersons’ willingness and readiness to respond to emergencies is crucial in this new context.
5. Timeliness with the HEARTS Act: This investigation is particularly timely in light of the recent passage of the HEARTS (Health Education, Awareness, Research, and Training in Schools) Act, which mandates schools to create cardiac emergency response plans, offer CPR and AED training, and ensure the accessibility of AEDs. One-quarter of this study’s participants were high school students whose voices are critical in shaping early training initiatives. As highlighted in the conclusion, “Early and continuous training, starting with middle and high school students, could help build a culture of action and prepare future laypersons to act promptly in cardiac emergencies”.

### 4.5. Limitations and Future Research and Interventions

This study has several limitations. The cross-sectional design limits the ability to assess changes in CPR knowledge and attitudes over time, particularly post-training, because no follow-up survey was administered. The transient nature of confidence and the potential decay of CPR skills over time may occur, which may affect participants’ willingness and ability to perform CPR or use AEDs long after the training. The sample consisted of highly motivated participants, which may not be representative of the general population. Although the sample mirrored the racial-ethnic makeup of the study neighborhood [52], there was a lower proportion of Non-Hispanic Black and Non-Hispanic White individuals available to make meaningful interpretations and could only be considered as hypothesis-generating evidence. There is a possibility that a small proportion of participants who answered ’Unsure’ to knowledge-based questions may not lack knowledge but could be uncertain because of unclear phrasing or unfamiliarity with medical terms. This misclassification may affect demographic groups with lower health literacy. Future studies should consider the potential bias when assessing knowledge gaps across racial or ethnic groups. Personal and cultural factors or prior experiences might influence participants’ motivations and attitudes toward administering CPR, which could vary across diverse communities. Further research using a mixed-methods approach should aim to explore these social and cultural factors. Finally, while the survey instrument was adapted from previous studies, the survey questions did not undergo validation studies, which could impact the reliability and generalizability of the responses.

Future research should involve longitudinal studies to evaluate the impact of training on long-term knowledge retention and behavior change, given the eventual goal is to improve preparedness and survival rates in high-risk communities. The non-clinical staff working in the high-resource outpatient sites had higher rates of training and were more educated about CPR/AED use but still carried the same level of concerns as low-resource laypersons. This finding suggests that training may need to be more frequently provided with a refresher and tailored to address their concerns. Early and continuous training, starting with middle and high school students, could help build a culture of action and prepare future laypersons to act promptly in cardiac emergencies [53].

## 5. Conclusions

This study characterizes laypersons’ attitudes toward CPR and AED use across various community settings. Regardless of the availability of the resources and prior training, confidence deficits in performing CPR and the fear of legal repercussions are layperson’s top concerns. Familiarity with the person needing CPR in any capacity was associated with increased willingness to perform CPR. Non-Hispanic Black participants often express greater and more specific concerns compared with their Non-Hispanic White counterparts. Will targeted, culturally sensitive interventions addressing these disparities, improve laypersons’ preparedness, and enhance survival rates for OHCA, is yet to be seen. Understanding and mitigating community-specific barriers may potentially foster a more inclusive and, thus, effective approach to emergency cardiovascular care.

## Figures and Tables

**Table 1:** Demographics and Characteristics of Laypersons in Low-Resource Communities.

Participants Characteristics (N = 669)	% (n)
Age	
<18 18–40 41–60 61–80 >81	26 (170) 33 (216) 23 (157) 16 (103) 2 (16)
Sex	
Male Female Other Decline to respond	31 (206) 67 (445) 1 (4) 1 (5)
Race	
American Indian	5 (34)
Asian/Pacific Islander	12 (77)
Black	24 (163)
White	56 (373)
Decline to respond	3 (22)
Ethnicity	
Hispanic/Latino	64 (426)
Non-Hispanic/Latino	32 (217)
Decline to respond	4 (26)
Education Status	
<High school	29 (188)
>High school	66 (425)
Decline to respond	5 (30)
Occupational Status	
Employed	41 (270)
Unemployed	16 (106)
Student	27 (175)
Homemaker	1 (10)
Retired	15 (97)
Have you ever learned CPR before?	
Yes	35 (230)
No	62 (409)
Unsure	3 (22)
Is Cardiac Arrest the same as a Heart Attack?	
Yes No Unsure	22 (146) 45 (292) 33 (212)
Have you ever learned how to administer an AED?	
Yes No Unsure	17 (117) 77 (504) 6 (37)
Do you know if there is an AED in the building where you work or study?	
I am certain there is an AED and know where it’s located I am certain there is no AED I’m not sure if there is an AED	22 (136) 16 (102) 62 (386)

**Table 2:** Concerns About Performing CPR in Low-Resource Communities: A Comparison Across Racial-Ethnicities and with High-Resource Communities.

Concerns	Low-Resource Communities (N = 632)	Hispanic (N = 402)	Non-Hispanic Black (N = 72)	Non-Hispanic White (N = 80)	*p*-Value	High-Resource Communities (N = 292)	*p*-Value
	% (n)	% (n)	% (n)	% (n)		% (n)	
Performing CPR incorrectly	67 (423)	64 (256)	79 (57)	64 (51)	0.03	63 (184)	0.3
Fear of harming the victim	56 (353)	49 (198)	75 (54)	61 (49)	<0.01	---	
Afraid of being sued if the person dies	53 (336)	53 (211)	61 (44)	55 (44)	0.4	47 (137)	0.2
Catching a disease	30 (212)	30 (120)	49 (35)	44 (35)	0.01	32 (92)	0.7
Being wrongfully accused of sexual harassment	24 (153)	23 (93)	17 (12)	23 (18)	0.5	---	
Somebody else would do it better	29 (184)	23 (94)	51 (37)	28 (22)	<0.01	23 (67)	0.3
Hurting myself while I provide CPR	13 (82)	12 (50)	15 (11)	14 (11)	0.7	12 (35)	0.9
Other	6 (37)	7 (30)	1 (1)	3 (2)	0.06	3 (8)	0.7

**Table 3:** Factors Associated with Willingness to Perform CPR in Low-Resource Communities: A Comparison Across Racial-Ethnicities and with High-Resource Communities.

Willingness Factors	Low-Resource Communities (N = 590)	Hispanic (N = 369)	Non-Hispanic Black (N = 68)	Non-Hispanic White (N = 76)	*p*-Value	High-Resource Communities) (N = 243)	*p*-Value
	% (n)	% (n)	% (n)			% (n)	
Knowing the person, other than family	67 (398)	63 (233)	82 (56)	70 (53)	<0.01	48 (116)	<0.01
Family member	55 (325)	56 (205)	57 (39)	54 (41)	0.9	39 (95)	<0.01
Medical professional	34 (198)	30 (111)	41 (28)	39 (30)	0.08	39 (95)	0.4
Age of patient	34 (203)	35 (130)	32 (22)	33 (25)	0.8	21 (51)	0.07
The responder has a physical disability	25 (147)	24 (89)	23 (16)	26 (20)	0.8	19 (45)	0.4
Gender of patient	20 (120)	21 (76)	16 (11)	20 (15)	0.7	10 (24)	0.2
Race/ethnicity of patient	14 (81)	12 (46)	22 (15)	7 (5)	0.03	8 (19)	0.5
Other	8 (46)	8 (28)	3 (2)	9 (7)	0.3	19 (47)	0.1

**Table 4:** Barriers Associated with Public Access Defibrillation in Low-Resource Communities: A Comparison Across Racial-Ethnicities.

Barriers.	Low-Resource Communities (N = 635)	Hispanic (N = 408)	Non-Hispanic Black (N = 66)	Non-Hispanic White (N = 80)	*p*-Value
	% (n)	% (n)	% (n)	% (n)	
Don’t know how to use	66 (417)	67 (273)	59 (39)	64 (51)	0.6
Will harm the other person	25 (158)	22 (90)	41 (27)	25 (20)	<0.01
Difficult to Use	17 (108)	13 (55)	29 (19)	20 (16)	<0.01
Think I am not allowed to use	15 (94)	14 (59)	28 (19)	8 (6)	<0.01
Will not help the person	4 (26)	4 (16)	9 (6)	1 (1)	0.06

**Table 5:** Associations between Concerns About Performing CPR in Low-Resource Communities and Racial-Ethnicities.

Concerns	Univariate Odds Ratio (95% CI)	*p*-Value	* Multivariate Odds Ratio (95% CI)	*p*-Value
Performing CPR incorrectly				
Non-Hispanic White Non-Hispanic Black Hispanic	Reference 2.2 (1.0–4.4) 1.0 (0.6–1.6)	0.03 0.9	Reference 2.5 (1.2–5.2) 1.0 (0.6–1.7)	0.02 0.9
Fear of harming the victim				
Non-Hispanic White Non-Hispanic Black Hispanic	Reference 1.9 (0.9–3.8) 0.6 (0.4–1.0)	0.07 0.05	Reference 2.0 (0.9–4.1) 0.5 (0.3–0.9)	0.06 0.01
Afraid of being sued if the person dies				
Non-Hispanic White Non-Hispanic Black Hispanic	Reference 1.3 (0.7–2.4) 0.9 (0.6–1.5)	0.4 0.7	Reference 0.9 (0.5–1.5) 1.2 (0.6–2.3)	0.7 0.6
Catching a disease				
Non-Hispanic White Non-Hispanic Black Hispanic	Reference 1.2 (0.6–2.3) 0.5 (0.3–0.9)	0.5 0.02	Reference 1.1 (0.6–2.2) 0.6 (0.4–1.0)	0.7 0.07
Being wrongfully accused of sexual harassment				
Non-Hispanic White Non-Hispanic Black Hispanic	Reference 0.7 (0.3–1.6) 1.0 (0.6–1.8)	0.4 0.9	Reference 0.7 (0.3–1.5) 1.0 (0.5–1.8)	0.3 0.9
Somebody else would do it better				
Non-Hispanic White Non-Hispanic Black Hispanic	Reference 2.8 (1.4–5.4) 0.8 (0.5–1.3)	<0.01 0.4	Reference 3.2 (1.6–6.5) 0.8 (0.5–1.5)	<0.01 0.5
Hurting myself while I provide CPR				
Non-Hispanic White Non-Hispanic Black Hispanic	Reference 1.1 (0.5–2.8) 0.9 (0.4–1.8)	0.8 0.7	Reference 0.9 (0.4–1.8) 0.9 (0.4–2.4)	0.8 0.9
Other				
Non-Hispanic White Non-Hispanic Black Hispanic	Reference 0.5 (0.1–6.1) 3.1 (0.7–13.4)	0.6 0.1	Reference 0.5 (0.0–5.2) 3.8 (0.9–16.5)	0.5 0.07

CPR: Cardiopulmonary Resuscitation; CI: Confidence Intervals; * The model was adjusted for the participant’s age, sex, education status, and prior CPR training status.

**Table 6:** Associations Between Factors Affecting Willingness to Perform CPR in Low-Resource Communities and Racial-Ethnicities.

Concerns	Univariate Odds Ratio (95% CI)	*p*-Value	* Multivariate Odds Ratio (95% CI)	*p*-Value
Knowing the person, other than family				
Non-Hispanic White Non-Hispanic Black Hispanic	Reference 2.0 (0.9–4.5) 0.7 (0.4–1.3)	0.08 0.3	Reference 2.3 (1.0–5.3) 0.8 (0.5–1.4)	0.04 0.5
Family member				
Non-Hispanic White Non-Hispanic Black Hispanic	Reference 1.1 (0.6–2.2) 1.1 (0.6–1.7)	0.7 0.8	Reference 1.2 (0.6–2.3) 1.1 (0.7–1.8)	0.6 0.7
Medical professional				
Non-Hispanic White Non-Hispanic Black Hispanic	Reference 1.1 (0.6–2.1) 0.7 (0.4–1.1)	0.8 0.1	Reference 1.3 (0.6–2.5) 0.7 (0.4–1.1)	0.5 0.1
Age of patient				
Non-Hispanic White Non-Hispanic Black Hispanic	Reference 1.0 (0.5–1.9) 1.1 (0.6–1.9)	0.9 0.7	Reference 1.0 (0.4–1.9) 1.2 (0.7–2.0)	0.8 0.5
The responder has a physical disability				
Non-Hispanic White Non-Hispanic Black Hispanic	Reference 0.9 (0.5–1.5) 0.9 (0.4–1.8)	0.7 0.7	Reference 1.0 (0.5–1.7) 0.9 (0.4–1.9)	0.9 0.7
Gender of patient				
Non-Hispanic White Non-Hispanic Black Hispanic	Reference 0.8 (0.3–1.8) 1.1 (0.6–1.9)	0.6 0.8	Reference 0.8 (0.3–1.8) 1.1 (0.6–1.9)	0.5 0.8
Race/ethnicity of patient				
Non-Hispanic White Non-Hispanic Black Hispanic	Reference 3.6 (1.3–10.5) 1.8 (0.7–4.7)	0.02 0.2	Reference 3.7 (1.2–10.8) 1.9 (0.7–5.3)	0.02 0.2
Other				
Non-Hispanic White Non-Hispanic Black Hispanic	Reference 0.3 (0.1–1.4) 0.8 (0.3–1.9)	0.1 0.6	Reference 0.3 (0.1–1.6) 0.8 (0.3–2.0)	0.2 0.6

CPR: Cardiopulmonary Resuscitation; CI: Confidence Intervals. * The model was adjusted for the participant’s age, sex, education status, and prior CPR training status.

## Data Availability

The original contributions presented in this study are included in the article. Further inquiries can be directed to the corresponding author.

## Appendix A. Community Development–Resuscitation Education, AED, and CPR Training (CD-REACT) Trainer Guide

There are three main elements of the ≈1-h training: Education, Demonstration, and Hands-on Skills Practice. Before the training: the coordinators from community affairs handle setup. Participants fill out a survey on their knowledge and beliefs regarding lifesaving interventions and receive a CPR Anytime Kit with Mannikin.

1. Education (15 min)
  - Introduce yourself to the audience and share what skills the audience will learn today.
    ○ If AV is available, we will play the American Heart Association’s CPR in Action video.
    ○ Ask the audience: What is cardiac arrest vs. heart attack? Do you have experience with giving CPR?
    ○ Top lines: Hands-Only CPR can triple the chance of survival. Chain of survival: identify cardiac arrest, call 911, start CPR, and administer an Automatic External Defibrillator (AED), if available, while you wait for paramedics. Goal: keep the brain alive; CPR pumps blood to the brain when the heart cannot.
2. Demonstration I: Hands-Only CPR (15 min)
  - Set the scene: You see a person pass out in a grocery store. Ask the audience: What do you do first?
    ○ Confirm unresponsiveness: Ask the person loudly, “Are you okay?”- NO RESPONSE
    ○ Check the breathing: if there is any abnormality in the breathing pattern → Call 911
    ○ Do not attempt to check the pulse to confirm—it is unreliable; you could be feeling your own pulse.
  - Demonstrate CPR and emphasize important elements:
    ○ Place your dominant hand on top of the non-dominant hand touching the chest and interlock fingers.
    ○ Find the area just below the center of the chest above the diaphragm. Keep your elbows locked and bend forward so your body weight can help you with compressions
    ○ 100 compressions per minute, two inches deep, allow the chest to come back to its normal state, uninterrupted for two minutes
    ○ NO more mouth-to-mouth breathing (not beneficial in adults). Avoid all CPR interruptions. The AHA still recommends CPR with compressions and breaths for infants, children, victims of drowning or drug overdose, and people who collapse due to breathing problems.
3. Hands-on Skills Practice (set to music) (20 min): Participants practice on their take-home CPR manikins, and the training team circles the room to help with form and answer questions. The manikins make a clicking noise when CPR is administered correctly.
4. Demonstration II: AED use (10 min): Return to the front of the room.
  - Ask the audience: Where can you find an AED? Airports, schools, restaurants, libraries, gyms. Do you know where an AED is in your workplace/school? If I open this device right now, will it shock me?
  - Ask a volunteer to come to the front and demonstrate AED use: place pads, provide shock, and resume CPR.
5. Call for a volunteer to run the ‘Chain of Survival’ (described above) and conclude (5 min).

FAQs:

○ What if I break someone’s ribs? Broken ribs can be repaired. You are saving a life.
○ Can you be sued for giving CPR to a stranger? No, NYS Good Samaritan Law protects you.
○ Is this training official CPR certification? No, but you will receive a Certificate of Completion.

## Appendix B. Community Development Resuscitation Education and CPR/AED Training (CD-REACT)

Anonymous Brief Awareness Questionnaire

Consent: I agree to participate in this anonymous and voluntary survey. I grant permission for the data to be used for informational purposes.	
□ Yes	□ No
1. Which of the following best describes your age?	
□ Less than 18 years old	□ Between 61–80 years old
□ Between 18–40 years old	□ More than 81 years old
□ Between 41–60 years old	
2. Which of the following best describes your sex?	
□ Male	□ Other __________________
□ Female	□ Decline to respond
3. Which of the following best describe(s) your race? (Mark all that apply):	
□ American Indian/Native American	□ Black
□ Asian or Pacific Islander	□ White
□ Decline to Respond	
4. Which of the following best describes your ethnicity? (Select only one):	
□ Hispanic or Latino	□ Decline to respond
□ Not Hispanic or Latino	
5. What is the highest grade or year of school you have completed?	
□ Less than high school	□ Decline to respond
□ More than high school	
6. What is your current working status?	
□ Employed—part or full time	□ Student
□ Unemployed	□ Homemaker
□ Retired	
7. Have you ever learned CPR (cardio-pulmonary resuscitation) before?	
□ Yes	□ Unsure
□ No	
8. Have you ever learned how to administer AED (Automatic External Defibrillator) before?	
□ Yes	□ Unsure
□ No	
9. Think about where you work or go to school. Do you know if there is an AED in the building?	
□ Yes, I am certain there is an AED and I know exactly where it’s located.	
□ I’m not sure if there is an AED.	
□ No, I am certain there is no AED.	
10. In your knowledge, is cardiac arrest the same as a heart attack?	
□ Yes	□ Unsure
□ No	
11. What is your primary motivation for taking this course today?	
□ General interest in learning	
□ To help a family member who might need it (child or ill)	
□ Work requirement	
□ To help/save a life in case of an emergency	
□ Work in healthcare or in a field with patient contact	
□ To teach others	
□ Other __________________	
12. What is the approximate number of people you are planning to teach CPR and AED use after taking this course using CPR Anytime Training Kits? _________________________________________	
13. In your opinion and experience, what are the main concerns people in the community might have about performing CPR? (Mark all that apply)	
□ Afraid to be sued if the person dies	
□ Being wrongfully accused of sexual harassment	
□ Catching a disease (e.g., during mouth-to-mouth breathing)	
□ Afraid of harming the person	
□ Hurting myself while I am doing CPR	
□ Somebody else would do it better	
□ Performing CPR incorrectly due to insufficient training	
□ Other (explain): __________________	
14. In your opinion and experience, which of the following factors might influence willingness to perform CPR on someone? (Mark all that apply)	
□ Knowing the person, in any capacity other than family	
□ Whether they are a family member or not	
□ Whether someone is a medical professional or not	
□ Age of the person in cardiac arrest	
□ Gender of the person in cardiac arrest	
□ Race/ethnicity of the person in cardiac arrest	
□ Whether person providing CPR has a physical disability	
□ Other (explain): __________________	
15. In your opinion and experience, what are some reasons for not using an AED on a stranger who collapses in public?	
□ An AED looks difficult to use.	
□ I do not think that I am allowed to use an AED.	
□ An AED will not help the person.	
□ I am afraid that an AED will harm the person.	
□ I don’t know how to use an AED.	
